# Mitochondria, calcium-dependent neuronal death and neurodegenerative disease

**DOI:** 10.1007/s00424-012-1112-0

**Published:** 2012-05-22

**Authors:** Michael R. Duchen

**Affiliations:** Department of Cell and Developmental Biology, University College London, Gower Street, London, WC1E 6BT UK

**Keywords:** Mitochondria, Intracellular calcium, Neurodegenerative disease, Glutamate excitotoxicity

## Abstract

Understanding the mechanisms of neuronal dysfunction and death represents a major frontier in contemporary medicine, involving the acute cell death in stroke, and the attrition of the major neurodegenerative diseases, including Parkinson's, Alzheimer's, Huntington's and Motoneuron diseases. A growing body of evidence implicates mitochondrial dysfunction as a key step in the pathogenesis of all these diseases, with the promise that mitochondrial processes represent valuable potential therapeutic targets. Each disease is characterised by the loss of a specific vulnerable population of cells—dopaminergic neurons in Parkinson's disease, spinal motoneurons in Motoneuron disease, for example. We discuss the possible roles of cell type-specific calcium signalling mechanisms in defining the pathological phenotype of each of these major diseases and review central mechanisms of calcium-dependent mitochondrial-mediated cell death.

## Introduction

The fine spatial and temporal organisation of intracellular calcium signals is fundamental to function in the CNS, perhaps more than in any other tissue. Signals are conveyed throughout the CNS by local changes in calcium concentration ([Ca^2+^]_c_) carrying information from neuron to neuron, between neurons and glia and even regulating local blood flow in relation to local activity. Local changes in [Ca^2+^]_c_ are the very stuff of our thoughts and sensations, our memories and dreams. Neuronal energy supplies are meanwhile entirely predicated on mitochondrial oxidative phosphorylation—neurons are almost exclusively dependent on mitochondrial ATP generation and have almost no capacity to upregulate energy supply through glycolysis when oxidative phosphorylation is compromised [33], making them especially vulnerable to mitochondrial dysfunction. Mitochondrial function and Ca^2+^ signalling are intimately linked, as the [Ca^2+^]_c_ signal is used as the major medium of a continuous subtle dialogue between the cytosol and its mitochondrial population that regulates energy homeostasis. It is the [Ca^2+^]_c_ signal that signals to mitochondria that more energy is required, and the [Ca^2+^]_c_ signal that mediates the mechanisms that deliver increased energy. [Ca^2+^]_c_ signals even tell mitochondria where to go in the labyrinthine maze of a complex neuron.

These mechanisms are so fundamental, that even subtle disturbances are likely to have far reaching functional consequences in disease. Defects in mitochondrial function are now implicated in a litany of major diseases throughout the CNS. Development of irreversible mitochondrial injury is almost certainly the critical step in the pathway to irreversible injury and cell death in the penumbra of an evolving stroke. Chronic defects in aspects of mitochondrial biology have been linked with most of the major neurodegenerative diseases. These include a range of genetic diseases—Huntington's disease and Friedreich's ataxia, some of the cerebellar ataxias, and heritable, familial forms of Parkinsons' disease, Motoneuron disease (also known as amyotrophic lateral sclerosis (ALS)) and Alzheimer's disease. These latter three diseases are unusual in this respect, as almost identical disease phenotypes are seen both as sporadic forms for which risk increases progressively with age, and as heritable familial forms, in which mutations of specific genes lead to almost identical disease phenotypes that usually occur in a younger age group of patients. The evidence for a mitochondrial pathophysiology is stronger in some of these diseases than in others, and a major challenge remains to define the processes that are primary mechanisms driving the pathophysiology, those which are contributors on the pathways to cell injury caused by other primary processes, and those that are epiphenomena, and ultimately to identify potentially useful therapeutic targets. These diseases are of huge importance as a major frontier in medical research, a major challenge to the pharmaceutical industry, and an enormous and terrifying threat to us all.

In this short essay, I will explore in particular the role of mitochondrial Ca^2+^ handling in neurons in the pathophysiology of these horrible and disabling diseases, with a focus on Ca^2+^-mediated cell death as a most extreme form of mitopathy that seems potentially tractable to therapeutic intervention, and that seems also to represent a ubiquitous form of cell injury that is recapitulated in many different disease contexts.

## Mitochondrial calcium handling in the CNS

One of the abiding and critical unanswered questions in all the major neurodegenerative diseases is the basis for the selectivity and specificity of the cellular targets of degeneration: the death of motoneurons in ALS, of Purkinje neurons in the cerebellar ataxias, of dopaminergic neurons of the substantia nigra in Parkinson's disease (PD) and of retinal ganglion cells in optic atrophy and Leber's hereditary optic neuropathy. This is most difficult in those genetic diseases for which mutations of proteins have been identified, as, in all of these, the mutant proteins are ubiquitously expressed while only the target cells are affected. This signals a search for mechanisms or features of the target cells that makes them especially vulnerable to the effects of the mutation, but it has to be said that the questions remain in the most part unsatisfactorily answered if answered at all.

There is growing evidence that the answer, at least in some cases, may be embedded in the cell-specific aspects of mitochondrial Ca^2+^ signalling—the overall theme of this special issue. This theme is perfectly exemplified in the CNS, where there is a striking diversity in the [Ca^2+^]_c_ signalling pathways employed by different cell populations. Indeed, it seems very plausible that the differences in expression of specific elements in the [Ca^2+^]_c_-signalling toolkit may be, at least in part, responsible for the cell type specificity of injury seen in most of the neurodegenerative diseases.

Calcium signalling in neurons is, in general, dominated by Ca^2+^ influx through voltage- and ligand-gated ion channels, with smaller modulations by intracellular signalling pathways, while [Ca^2+^]_c_ signalling in glial cells is dominated by Ca^2+^ release from ER using IP3 as a second messenger system. This very crude classification leads to further sub-specialisations, depending on the classes of ion channels expressed by the neurons, neuronal architecture, cell size, Ca^2+^ buffering power and patterns of excitability. Thus, the specific Ca^2+^ signalling physiology of a cerebellar Purkinje cell is radically different from that of the neighbouring cerebellar granule cell, although both are ‘neurons’, reflecting differences in size and complexity and the requirement for local amplifying signalling mechanisms. Spinal motoneurons differ in many respects from many other cell types in their large volume, their enormously long axons and in their expression of Ca^2+^ permeant ‘AMPA’ subclass of glutamate receptors [13], combined, apparently with a relatively low Ca^2+^ buffering power [29, 54]. The dopaminergic neurons of the substantia nigra that selectively degenerate in PD have an unusual physiology in terms of their repetitive rhythmic excitability driven by pacemaking L-type Ca^2+^ channels [64]. Amongst the glia, astrocyte, microglial and oligodendrocyte Ca^2+^ signalling are all different, still to be fully explored and beyond the scope of this review.

## Calcium regulates local mitochondrial metabolism

While much of the early literature demonstrating pathways for physiological mitochondrial Ca^2+^ accumulation and extrusion arose from studies of isolated mitochondria from liver or heart, it turns out that many of the earliest demonstrations of physiological mitochondrial Ca^2+^ uptake came from neuronal systems. A recurrent theme throughout this special issue is the activation of mitochondrial metabolism through the stimulation of the tricarboxylic acid (TCA) cycle by a rise in the matrix Ca^2+^ concentration [48]. One of the earliest demonstrations that a rise in Ca^2+^ concentration can increase mitochondrial oxidative phosphorylation was made in the photoreceptor, albeit from that strangest of creatures, the horseshoe crab [24] nearly a quarter of a century ago. Around the same time, Thayer and colleagues showed that during the time period immediately following (but not before) generation of Ca^2+^ signals through physiological pathways (although admittedly not to stimuli that could be called very physiological) in sensory neurons, the collapse of mitochondrial membrane potential by an uncoupler caused a large Ca^2+^ signal, presumably due to the release of Ca^2+^ from mitochondria, inferring that the mitochondria had been Ca^2+^ loaded by the prior Ca^2+^ increase, i.e. the mitochondria were accumulating Ca^2+^ during a physiological stimulus, a controversial issue at that time [66]. This was followed by our own work, also in sensory neurons, showing changes in mitochondrial function—a brief transient mitochondrial depolarization associated with mitochondrial Ca^2+^ uptake across the inner membrane, followed by prolonged, Ca^2+^-dependent increase in NADH—following depolarization-induced Ca^2+^ signals also in sensory neurons, again with the inference that mitochondria must be taking up Ca^2+^ under these physiological conditions. The interpretation was that mitochondria were taking up Ca^2+^, which activated the TCA cycle, increasing NADH and driving increased oxidative phosphorylation [22]. Even in complex preparations of neuronal networks, such as the hippocampal slice preparation, stimulation of synaptic pathways led to Ca^2+^-dependent changes in NADH fluorescence suggesting direct modulation of metabolism by Ca^2+^ signals [38, 60]. Thus, the Ca^2+^ signals that are essential for synaptic transmission and therefore for transmission of information throughout the CNS are transmitted to the mitochondria where it is assumed that Ca^2+^ modulates mitochondrial metabolism as described elsewhere—with upregulation of the TCA cycle, of the ATP synthase, of the aspartate carrier [56] and presumably with a resultant increase in the supply of ATP. I am intentionally cautious here, as a formal direct demonstration of increased ATP generation in neurons in response to synaptic stimulation is lacking as yet, although this has been demonstrated in other cell types [37].

If mitochondria take up Ca^2+^, it follows that they have a potential to act as local Ca^2+^ buffers. The effective impact is a quantitative matter—how densely packed are the mitochondria and what is their capacity to accumulate Ca^2+^? There are good data to show that mitochondrial Ca^2+^ uptake has a sufficient impact on local Ca^2+^ signalling to help shape synaptic transmission, both at the neuromuscular junction [18, 19] and at the giant synapse of the calyx of Held [8]. We also showed that mitochondrial Ca^2+^ uptake has sufficient buffering power to determine the rate of propagation of Ca^2+^ signals as waves through astrocytes [9]. As the subtle shaping and the spatial and temporal characteristics of Ca^2+^ signals are essential in shaping neuronal activity, it is very tempting to speculate that this subtle impact will be altered in disease states in which mitochondrial function is disturbed, having a disproportionate impact on CNS function. This remains hard to study directly and remains largely speculative.

## Calcium directs the mitochondrial traffic

Calcium signals also seem to play a critical role in directing the mitochondrial traffic in complex neuronal networks. Mitochondria must be continually refreshed with new mitochondrial protein (the vast bulk of which is encoded by nuclear DNA), and must then be transported sometimes very long distances along axonal routes to reach the synaptic terminals where they are most needed. Travelling mitochondria have to find their way through complex dendritic trees and up and down long axons in a cell that may be effectively a metre long. Trafficking in general is beyond the scope of this review, but in principle involves the interaction between proteins on the outer mitochondrial membrane, including mitofusin 2 (MFN2) and the Ca^2+^-dependent Miro1. The local positioning of mitochondria near points of high energy demand seems functionally important and appears to be largely orchestrated by Ca^2+^. Thus, a local rise in [Ca^2+^]_c_ interacts with the outer mitochondrial protein Miro, inhibiting trafficking through the kinesin motor KIF5, so that mitochondria stop where they are, and remain localized at areas where [Ca^2+^]_c_ is high—presumably the places where energy demand will be highest and where local Ca^2+^ buffering is also likely to be important [44]. Defects in axonal mitochondrial transport lead to long tract dysfunction, such as the Charcot Marie Tooth peripheral sensory neuropathy (type 2A) that involves a defect in the protein MFN2 which interacts with Miro1 and 2 [50]. Defects in mitochondrial trafficking proteins have also been associated with Parkinson's disease. The kinase PTEN induced kinase 1 (PINK1) is one of an increasingly large family of proteins for which mutations are associated with familial forms of PD (see also below). PINK1 expression on the outer mitochondrial membrane increases in depolarized mitochondria, and recruits the ubiquitin ligase Parkin to mitochondria, promoting mitochondrial autophagy (mitophagy). It turns out that PINK1 phosphorylates Miro, activating its proteosomal degradation through the PINK1-dependent recruitment of Parkin. It is proposed that degradation of Miro limits mitochondrial movement, and might serve to ‘quarantine damaged mitochondria’, prior to their removal by autophagy with the suggestion that mutations in these proteins will impair the processes that segregate and remove dysfunctional mitochondria, allowing their accumulation and so leading to progressive neurodegeneration [50]. Defective axonal transport of mitochondria has also been associated with Motoneuron disease (discussed briefly below).

## Roles of mitochondria in calcium-dependent neurotoxicity

A decisive element in the neuronal signalling machinery is the expression of the glutamate receptor families. Of these, two are highly Ca^2+^ permeable—the NMDA receptor and the Ca^2+^-permeant AMPA receptor. These play a crucial role in regulating Ca^2+^ influx related to synaptic activity and modulation, but they also create a precarious balancing act for neuronal Ca^2+^ homeostasis. It has been apparent for many years that prolonged exposure of neurons in culture to high concentrations of glutamate leads to Ca^2+^-dependent cell death—a phenomenon described as excitotoxicity [16]. Not only was it clear that glutamate caused Ca^2+^-dependent toxicity but it also seemed that not all Ca^2+^ is the same—the toxicity of Ca^2+^ depends on the route of entry. Michael Tymianski coined the term ‘the source specificity’ of Ca^2+^-dependent toxicity, showing that Ca^2+^ influx into hippocampal neurons via L-type voltage-gated Ca^2+^ channels was innocuous, while a similar global Ca^2+^ load (measured using radiolabelled calcium to avoid artefacts due to dye saturation) arriving in the cell through NMDA receptors was lethal [67]. His group later went on to show in a most elegant series of experiments that the source specificity arises from the colocalisation of NMDA receptors with nNOS, coupled through the post synaptic density protein, psd-95 [58]. The Ca^2+^-dependent nNOS was thus exposed to local microdomains of very high [Ca^2+^]_c_ as the ion enters through the Ca^2+^-permeant NMDA channels, generating NO which in combination with the [Ca^2+^]_c_ signal is toxic to the cells. Importantly, cells could be rescued by nNOS inhibition or by decoupling the nNOS from the NMDA receptor.

This process is probably not an experimental oddity of neurons in tissue culture but is thought to occur in the brain under pathological conditions. The clearest role is in ischaemic disease in the CNS—in stroke for example—where glutamate can rapidly accumulate in the extracellular space to toxic concentrations of hundreds of micromolar. It is believed that this mechanism is likely responsible for the progression of a stroke over the first few days after an ischaemic episode, extending the areas of brain injury beyond the core ischaemic zone into a penumbra. What completed the circle very nicely was the demonstration by Tymianski and colleagues that decoupling nNOS from the NMDA receptors using small peptides rendered membrane permeant with the HIV TAT sequence rescued CNS tissue in an in vivo stroke model [1].

The source specificity of glutamate-induced neuronal damage has been refined more recently to suggest that excessive activation of extrasynaptic receptors may induce toxic Ca^2+^ loads and mitochondrial Ca^2+^-dependent injury, whilst activation of synaptic receptors tends to cause smaller Ca^2+^ elevations that play a prosurvival role [61], suggesting that the balance between these two pathways may be critical in some disease models, in which glutamate ‘overspill’ from synaptic release, glutamate release from glial cells by reversal of the glutamate transporter, or impaired glutamate clearance from synaptic clefts might lead to overactivation of extrasynaptic receptors, causing cell injury.

**Fig. 1:**
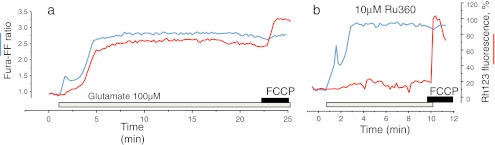
Simultaneous measurements of [Ca^2+^]_c_ (*blue trace*) and of Δψ_m_ (*red trace*) from single hippocampal neurons in response to toxic glutamate exposure. Δψ_m_ was measured using the dye rhodamine 123 which was used in the so-called ‘dequench’ mode, in which mitochondrial depolarization causes an increase in fluorescence signal. [Ca^2+^]_c_ was measured using fura-FF, a low affinity ratiometric [Ca^2+^]_c_ indicator. **a** The response to glutamate shows a stereotypical pattern: the [Ca^2+^]_c_ increases transiently followed by variable degree of recovery, followed by a progressive increase—referred to as delayed [Ca^2+^]_c_ deregulation. Δψ_m_ changes only slightly during the initial phase, but then the mitochondria show a progressive and complete depolarization (addition of uncoupler which depolarises completely has no further effect) that is exactly synchronous with DCD. **b** Following preincubation with Ru360, inhibitor of the mitochondrial uniporter, the [Ca^2+^]_c_ signal was essentially unchanged, but mitochondrial depolarization was suppressed, suggesting that mitochondrial [Ca^2+^]_c_ uptake is an essential step on the pathway to [Ca^2+^]_c_-mediated cell death [2]

So, where do the mitochondria come in? One of the major features of [Ca^2+^]_c_-dependent toxicity is the ‘delayed deregulation of [Ca^2+^]_c_ homeostasis’ (delayed [Ca^2+^]_c_ deregulation, or DCD)—a secondary rise in [Ca^2+^]_c_ that can be delayed by anything from 1 or 2 to 10 min after the initial exposure to glutamate (Fig. 1a). Simultaneous measurements of mitochondrial membrane potential and [Ca^2+^]_c_ showed that the secondary [Ca^2+^]_c_ deregulation occurs in synchrony with a [Ca^2+^]_c_-dependent collapse of mitochondrial membrane potential (Δψ_m_) [69] (Fig. 1a). The loss of Δψ_m_ was preventable using nNOS inhibitors as was the DCD, and indeed, mitochondria were sensitised to otherwise innocuous [Ca^2+^]_c_ loads by NO donors [39], suggesting a direct link between the events at the plasma membrane and routes to mitochondrial toxicity.

Key questions then are what are the mechanisms of DCD and of loss of mitochondrial membrane potential? Once mitochondrial potential is lost in a neuron, that cell is destined to die as neurons have almost no capacity to increase glycolytic ATP once oxidative phosphorylation is lost [33], and so neurons with depolarized mitochondria will undergo bioenergetic collapse and die. It has been generally assumed that the loss of mitochondrial membrane potential is triggered by mitochondrial Ca^2+^ accumulation (‘overload’), a state where influx into the mitochondria overwhelms matrix Ca^2+^ buffering power and the capacity of the sodium–calcium exchange efflux pathway. Direct measurements of increased intramitochondrial free Ca^2+^ during this process have proven remarkably difficult—transfection of neurons with genetically encoded Ca^2+^ reporters is not easy, and large intracellular pH shifts associated with the massive movement of Na^+^ and Ca^2+^ across the membrane introduce major artefacts into most measurements. Using small molecule sensors such as rhod family dyes have also failed in most people's hands, as dye loading invariably is not restricted to the mitochondria and cytosolic [Ca^2+^]_c_ rises so high that the cytosolic component overwhelms the mitochondrial specific signal, which is probably saturated anyway. It has been shown that brief exposure to uncoupler around the time of glutamate application can be protective [62]. The logic was that the uncoupler, by collapsing the potential at the crucial time, prevents mitochondrial Ca^2+^ uptake. Even though the change in [Ca^2+^]_c_ was if anything enhanced, cell viability was improved, arguing that mitochondrial Ca^2+^ uptake plays a critical role in driving cell death. This is a tricky experiment as uncouplers can only too easily kill the cells, and so the timing is critical.

We showed relatively recently that inhibition of the Ca^2+^ uniporter using Ru360 prevented the loss of mitochondrial membrane potential and decoupled DCD and mitochondrial depolarization, as DCD proceeded even though mitochondrial potential was preserved [2] (Fig. 1b). This is perhaps the closest so far to a direct demonstration of a role for mitochondrial matrix Ca^2+^ overload as a determinant of mitochondrial dysfunction (see also [21]). It would be ideal to know whether this is protective against cell death, but Ru360 is highly unstable and tricky to use—it is oxidised rapidly after exposure to room air, and only ‘works’ for about 20–30 min after addition to the cells, making this an impossible experiment, and as yet, the appropriate tools to address this question directly are not available.

## The mitochondrial permeability transition pore

Demonstrations of mitochondrial depolarization following a rise in [Ca^2+^]_m_ and dependent on nitrosative stress immediately flags involvement of the mitochondrial permeability transition pore (mPTP) for any mitochondrial biologist. Therefore it is timely to introduce the permeability transition pore into this text. This is an extraordinary phenomenon, which seems to underlie Ca^2+^-dependent mitochondrial cell death in most tissues [31]. First described in a series of remarkable papers by Haworth and Hunter [32, 34, 35], this sudden loss of the mitochondrial permeability barrier following additions of Ca^2+^ or prooxidants was treated for a long while as an artefact of isolated mitochondrial preparations. Much later, it was shown that the permeability increase is due to the opening of a large conductance pore in the inner mitochondrial membrane large enough to admit deoxyglucose into the matrix and is not simply a disruption of the membrane [30]. It has been suggested that the pore is generated by a transformation of membrane proteins with other physiological roles into a pore-forming configuration—favoured candidates included the adenine nucleotide translocase (ANT), a protein which can switch to a pore-forming conformation in the presence of high Ca^2+^ [57] and pore opening is modulated by drugs which bind to the ANT, and the voltage-dependent anion channel (VDAC) in the outer mitochondrial membrane. However, recent experiments on tissues from an ‘ANT and VDAC knockout mice’ have thrown question marks over this model [6, 42] leaving considerable uncertainty about the molecular identity of the mPTP. What is clear and unambiguous is that the pore opening is regulated by the matrix peptidyl prolyl *cis–trans* isomerase, the protein cyclophilin D [5, 65]. This is particularly important, as it binds to cyclosporine A (CsA), which prevents pore opening. CsA has become the benchmark for mPTP opening and is now being used in clinical trials for mPTP involvement (along with other mPTP inhibitors) in various pathologies [28, 49].

The role of the mPTP in cell death during ischaemia and reperfusion in the heart is clear and unambiguous. In the CNS, the storey has been less straightforward. CsA proved to be protective in stroke models [26] and in culture models of glutamate toxicity. The problem is that CsA binds to all cyclophilins, and these include cytosolic proteins that regulate calcineurin, which in turn mediates the Ca^2+^-dependent activation of nNOS [20]. Given the role of nNOS described above, this undermines the interpretation of experiments that demonstrate any protective action of CsA and, in the absence of other more specific pharmacological agents, has made it difficult to assign an unambiguous role for mPTP in glutamate toxicity or stroke. After generation of the CypD knockout mouse, it was soon shown that infarct size was reduced in the CypD knockout, arguing for a role for the PTP in stroke and cell death in the CNS [5]. To our surprise, however, the loss of mitochondrial potential in response to glutamate was not notably altered in cell models from the CypD knockout mouse [2], arguing that the initial loss of potential cannot be a simple consequence of mPTP opening. What we did find was that the loss of potential that was normally irreversible became reversible in the CypD knockout, suggesting that during a prolonged mitochondrial depolarization there was a delayed transition from depolarized mitochondria to mPTP opening [2].

## A role for PARP?

**Fig. 2:**
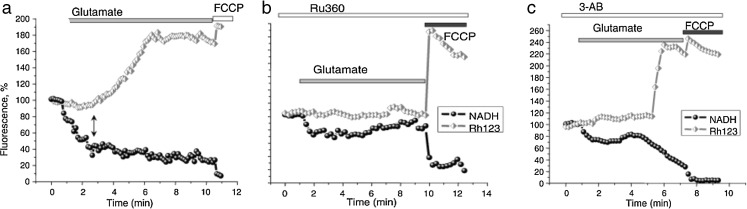
Simultaneous measurements of Δψ_m_ and of NADH autofluorescence from single hippocampal neurons. **a** Glutamate exposure caused a rapid oxidation of NADH causing a decrease in signal (as NAD^+^ is not fluorescent). Once the NADH oxidation reaches a nadir, Δψ_m_ started to fall (an increase in fluorescence signal). Final addition of uncoupler, FCCP, which causes maximal oxidation of mitochondrial NADH had no effect, suggesting that the NADH pool was already mostly oxidised. **b** Both the changes in Δψ_m_ and in NADH were largely inhibited following preincubation with Ru360 and were profoundly delayed by the PARP inhibitor 3-AB (**c**). Modified from [2]

**Fig. 3:**
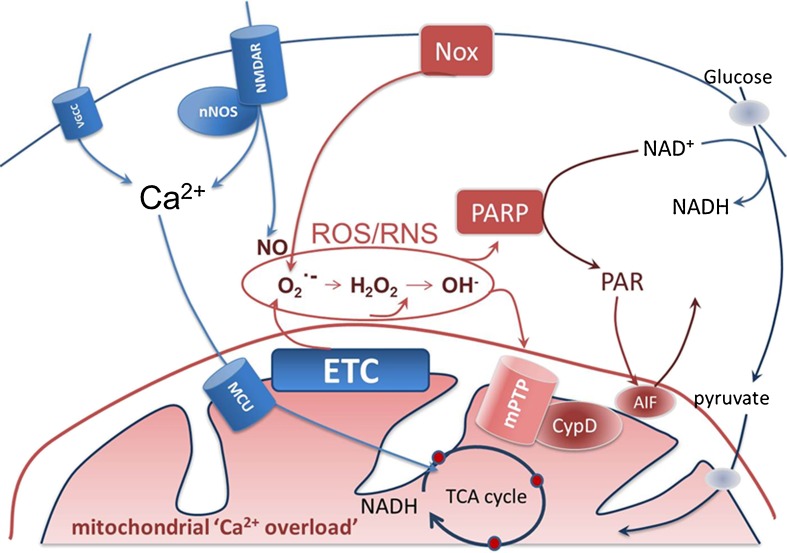
Scheme of pathways involved in glutamate-induced excitotoxicity. Calcium influx through voltage-gated or NMDAR-gated channels is followed by mitochondrial Ca^2+^ influx through the mitochondrial calcium uniporter (*MCU*). While the physiological consequence of raised intramitochondrial [Ca^2+^] is an increased activity of the three rate limiting enzymes of the TCA cycle, pathological and prolonged Ca^2+^ influx leads to mitochondrial Ca^2+^overload. NMDAR-mediated Ca^2+^ influx is closely coupled to the generation of NO by nNOS; raised Ca^2+^ may activate the NADPH oxidase (*Nox*), while mitochondrial Ca^2+^ overload may also increase generation of superoxide by the electron transport chain (*ETC*). Nitrosative or oxidative stress arising either from the ETC or from Nox activation may cause overactivation of PARP. PARP consumes NAD + to form PAR polymers, causing depletion of NAD^+^, failure of glycolysis and so failure of mitochondrial substrate supply. This culminates in the loss of Δψ_m_, ATP depletion and cell death. The PAR polymers generated by PARP may also cause release of AIF which amplifies cell death following its translocation to the nucleus

This leaves the question of the cause of the initial mitochondrial depolarization caused by glutamate excitotoxicity. In the late 1990s, evidence from several labs suggested that glutamate toxicity engaged the enzyme PARP-1 [23, 43, 46]. Poly (ADP ribose) polymerase-1 is a DNA repair enzyme that is activated by single strand DNA breaks, usually the result of oxidative stress. Activation of PARP uses NAD^+^ to generate polymers—poly (ADP ribose), or PAR polymers—which are relatively shortlived as these are rapidly degraded by the enzyme PAR Glycohydrolase [63]. It appears that PAR polymers can release apoptosis inducing factor (AIF) from mitochondria, suggesting that cell death may be mediated by AIF translocation to the nucleus and a ‘caspase-independent apoptosis’—a form of cell death that has been termed ‘parthanatos’ [70]. This is a fascinating model, but remains somewhat controversial. Several groups have shown that PARP-1 is hyperactivated by glutamate toxicity, but details of the pathways to cell death are less clear. Thus, it has been proposed that activation of NMDA receptors can activate the enzyme NADPH oxidase, generating oxidative stress that then activates PARP [10]. The activation of PARP consumes NAD^+^, prevents cell metabolism and the cells die. AIF may be released but seems immaterial, as the cells will die through bioenergetic collapse [3, 4]. In this model, it is hard to identify any specific role for mitochondrial Ca^2+^ uptake or to see why preventing mitochondrial Ca^2+^ uptake should be protective. Another group have suggested that the oxidative stress that activates PARP-1 is the result of mitochondrial Ca^2+^ uptake—i.e. mitochondria take up Ca^2+^, the matrix Ca^2+^ overload generates excessive free radical species, this causes activation of PARP-1 which then depletes NAD^+^ and the cells die [21]. Our own data suggest that mitochondrial Ca^2+^ uptake is indeed critical for PARP-1 activation, and suggest that PARP-1 activation causes the loss of mitochondrial membrane potential by inhibition of glycolysis and so depletion of mitochondrial substrate supply [2]. Thus, we found that application of glutamate to neurons causes a remarkably rapid decrease in the autofluorescence of NADH that precedes the loss of potential (Fig. 2a). Importantly, loss of NADH and mitochondrial depolarization were prevented by Ru360, (Fig. 2b) arguing that mitochondrial Ca^2+^ uptake is an essential step on the pathway to PARP activation and to cell death [2]. Pharmacological inhibition of PARP delayed both the NADH oxidation and the loss of potential (Fig. 2c). Remarkably, the mitochondrial membrane potential could be restored using substrates that bypass glycolysis—pyruvate or methyl succinate for example, suggesting that mitochondrial depolarization occurs as a failure of substrate supply as NAD^+^ is consumed and inhibits glycolytic supply of pyruvate. Again, it was hard to see an essential role for AIF, as the loss of potential condemns these cells to die. So the precise role for AIF remains a little unclear. What again brings the data full circle is the recent demonstration that small molecule PARP inhibitors are protective in a stroke model, putting the mechanism into a bigger context of brain injury and stroke [51]. The proposed full pathways of excitotoxicity are illustrated schematically in Fig. 3.

## Mitochondrial calcium handling and Parkinson's disease

Similar mechanisms of Ca^2+^-induced cell injury have been implicated in the pathogenesis of PD. The dopaminergic cells of the substantia nigra that degenerate in PD have an unusual electrical feature as pacemaking neurons, in which electrical activity is driven largely by L-type Ca^2+^ channels [64]. It has been argued that this Ca^2+^-mediated activity sensitises the pacemaking dopaminergic cells to injury, to the extent that inhibition of L-type Ca^2+^ channels (a condition in which electrical activity is taken over by Na^+^-mediated activity) can protect the cells against injury by toxins such as 6-(OH)DA [15]. The specific role of downstream pathways involving mitochondrial Ca^2+^ handling are perhaps less clear. The Ca^2+^ load appears to increase mitochondrial free radical generation, which in normal mice induces a protective mechanism through a mild uncoupling mechanism mediated by the uncoupling proteins UCP3 and 4, with the suggestion that this might reduce mitochondria free radical generation and so serve a protective role. Thus, a modest mitochondrial depolarization was inhibited by the UCP antagonist genipin. Of greatest interest perhaps was the loss of this protective mechanism by knockout of the protein DJ-1 and protection by overexpression of DJ-1. DJ-1 (also known as PARK7) is a protein in which mutations are associated with early onset familial form of PD. The data presented by the Surmeier group suggested that PTP and CypD were not involved, but the mechanism of cell death remains unclear [15]. Interestingly, other data also point to a role for PARP in PD but again the specific link between mitochondrial dysfunction, mitochondrial Ca^2+^ uptake, Ca^2+^ signalling and PARP does not seem to have been made [36, 45].

Amongst the neurodegenerative diseases, the case for a role of mitochondrial dysfunction in PD is probably the strongest. Several models involving toxic damage to the CNS, using the mitochondrial complex I inhibitor, rotenone, the drug derived compound MPTP, which also blocks complex I, and 6(OH)-DA, all cause mitochondrial and selective damage to dopaminergic neurons. Coupled with findings of Complex I dysfunction in SN from post mortem material from PD patients, it seems likely that this points to a major role for mitochondrial bioenergetic dysfunction in PD [59]. Huge strides have been made in the genetics of familial PD over recent years, with the identification of a string of proteins in which mutations cause early onset PD (for a review see: [25]). Remarkably, the picture that is emerging from these studies again points towards a pivotal role for mitochondrial dysfunction, as almost all of these proteins seem either to associate with mitochondria or to lie on mitochondrial associated pathways. Detailed discussion of this literature and the proteins involved is outside the scope of this essay, and I refer the reader to excellent recent reviews for further detail [11, 12]. However, the notion that the underlying defect may involve the failure of appropriate removal of dysfunctional mitochondria allowing the slowly progressive accumulation of mitochondrial defects as the basis for a neurodegenerative disease [53] seems quite appealing, even though these proteins are ubiquitous and so leaves us once again with the question why the dopaminergic SN neurons should be especially vulnerable.

In our own work, we addressed the functional bioenergetic consequences of a mutation of the protein PINK1 and its consequences for the neuronal vulnerability to calcium-mediated injury [27]. In PINK1 deficient cells, mitochondrial potential was reduced and was largely maintained by the ATPase running in reverse mode. This state was reversed by the provision of substrates for complex I and II (pyruvate or methyl succinate), respectively, suggesting a defect in substrate supply as a key mechanism underlying the bioenergetic defect. We also found that mitochondrial Ca^2+^ efflux was profoundly delayed in these cells, suggesting impaired Na^+^/Ca^2+^ exchange in mitochondrial Ca^2+^ homeostasis. The functional consequence of this defect was to make mitochondria far more vulnerable to Ca^2+^ overload and to dramatically reduce the effective threshold for mPTP opening, leading to Ca^2+^-dependent cell death. At present, it is not clear how to match these findings with our understanding of a role for PINK1 as a regulator of mitochondrial autophagy, unless we propose that the bioenergetic deficiencies are a consequence of failure of autophagic removal of damaged mitochondria. However, the observations provide a link between the Ca^2+^ models of PD dysfunction and the emerging literature pointing towards mitochondrial dysfunction as a pathophysiological mechanism in PD.

In Huntington's disease, there are also data suggesting that the pathology involves increased mitochondrial sensitivity to Ca^2+^ leading to a decreased threshold for mPTP opening. Huntington's disease is caused by an expansion of exonic CAG triplet repeats in the gene encoding the Huntingtin protein (Htt) that appear to be toxic. Mitochondria isolated from the brains of transgenic mice expressing mutant Htt showed depolarisation at lower Ca^2+^ loads than controls. Remarkably, this observation could be replicated in normal mitochondria exposed to long polyglutamine repeats, suggesting some kind of direct sensitising effect of the mutant protein [55].

## Motoneuron disease

The other major neurodegenerative disease that I must touch on is Motoneuron disease, or ALS. This is an appalling rapidly progressive and fatal disease involving the selective degeneration of spinal motoneurons. As in PD, there are rare familial forms of the disease which have been associated with mutations of superoxide dismutase (mSOD-1) and more recently with mutations of the protein vesicle associated membrane protein associated protein B (VAPB) [52]. The transgenic mutant mouse expressing the G93A mSOD-1 mutation develops selective degeneration of spinal motoneurons, and so represents a very appealing model to study ALS. There is always a major issue with using the mutations associated with any of the familial forms of major neurodegenerative diseases in an attempt to understand the sporadic disease which affects the majority of ALS patients. Familial ALS (fALS) associated with the SOD-1 mutation accounts for only a fraction of the total number of patients suffering this horrible disease: the incidence of familial ALS is usually estimated at 5–10 % of all patients with ALS, of which only one fifth express the SOD-1 mutation—i.e. this mutation accounts for about 2 % of all ALS patients, and yet it is the major model that is studied, if only for want of an adequate model of the sporadic disease. It is hard to know how much these studies will really tell us about the sporadic disease, but there is no doubt that it does tell us a great deal about general principles governing pathophysiological mechanism.

There is a substantial literature suggesting that motoneuron death in the mSOD-1 G93A genetic model of fALS is associated with mitochondrial dysfunction and some evidence linking changes in Ca^2+^ and mitochondrial Ca^2+^ overload. Thus, motoneurons express Ca^2+^-permeable AMPA receptors and also appear to have a relatively low Ca^2+^ buffering capacity. Exposure of motoneurons to AMPA caused increased ROS generation, loss of mitochondrial potential and cell death [14]. Furthermore, motoneurons expressing the G93A SOD-1 mutation were protected by overexpression of the Ca^2+^-buffering protein, parvalbumin [68], and also by knockdown of cyclophilin D [47], together pointing towards mPTP-related mechanism of glutamate-induced injury, mediated by AMPA receptors rather than the NMDA receptors that drive glutamate toxicity in cortical and hippocampal neurons. Interestingly, it seems that neurodegeneration in the fALS model is initiated at nerve terminals and tracks back along the axons. Gathering evidence suggests that Ca^2+^-mediated mitochondrial toxicity may be associated with repetitive stimulation in peripheral motoneuron axons [7], perhaps operating exactly the same mechanisms discussed above. Indeed, there is some evidence for a role of PARP in ALS as well, suggesting that elements of this Ca^2+^-dependent mitochondrial cell death pathway are recapitulated in different diseases, perhaps shaped by the specific physiology of each cell type [17, 40, 41].

There is also substantial evidence pointing to a disturbance in mitochondrial axonal transport in fALS. Thus, the VAPB mutation also seems to target Ca^2+^ and mitochondria but relating to mitochondrial transport in axons. A general defect in retrograde axonal transport has been described in the axons of G93A mutant mice, preceding the onset of symptoms, while a selective defect of anterograde axonal mitochondrial transport and a decrease in axonal mitochondrial content has also been described in the G93A mutant [52]. The VAPB mutation (VAPB P56S) also causes a selective defect in anterograde transport of mitochondria in axons associated with a rise in local [Ca^2+^]_c_, probably related to ER disruption (VAPB is an ER protein). The local elevation of the [Ca^2+^]_c_ signal regulates Miro1-mediated axonal mitochondrial transport (see above) leading to impaired mitochondrial trafficking and distribution. It is not clear why this defect should selectively affect motoneurons, nor why it should lead ultimately to motoneuron degeneration.

## Conclusions

Mitochondrial dysfunction is clearly implicated in the pathogenesis of the major neurodegenerative diseases and plays a critical role as a determinant of irreversible cell injury following stroke. Increasing evidence suggests that specialisations of Ca^2+^ signalling pathways in different cell types may play a major role in shaping the patterns of cell loss in each of the major neurodegenerative diseases. Understanding these pathways brings the promise of defining new potential therapeutic targets for these dreadful diseases.
